# Coalescence and mixing dynamics of droplets in acoustic levitation by selective colour imaging and measurement

**DOI:** 10.1038/s41598-023-46008-z

**Published:** 2023-11-10

**Authors:** Kota Honda, Kota Fujiwara, Koji Hasegawa, Akiko Kaneko, Yutaka Abe

**Affiliations:** 1 Graduate School of Science and Technology, University of Tsukuba, Tsukuba, 305-8573 Japan; 2 Department of Mechanical Engineering, Kogakuin University, Tokyo, 163-8677 Japan; 3 Institute of Systems and Information Engineering, University of Tsukuba, Tsukuba, 305-8573 Japan; 4 Professor Emeritus, University of Tsukuba, Tsukuba, 305-8573 Japan

**Keywords:** Fluid dynamics, Imaging techniques

## Abstract

Acoustic levitation is well-suited to ‘lab-on-a-drop’ contactless chemical analysis of droplets. Rapid mixing is of fundamental importance in lab-on-a-drop platforms and many other applications involving droplet manipulation. Small droplets, however, have low Reynolds numbers; thus, mixing via turbulence is not possible. Inducing surface oscillation is effective in this regard, however, the relationship between internal flow and mixing dynamics of droplets remains unclear. In this study, we conducted a set of simultaneous optical measurements to assess both the flow field and the distribution of fluid components within acoustically levitated droplets. To achieve this, we developed a technique to selectively separate fluorescent particles within each fluid, permitting the measurement of the concentration field based on the data from the discrete particle distribution. This approach revealed a relationship between the mixing process and the internal flow caused by surface oscillation. Thus, the internal flow induced by surface oscillation could enhance droplet mixing. Our findings will be conducive to the application and further development of lab-on-a-drop devices.

## Introduction

‘Lab-on-a-drop’ platforms present the possibility of performing contact-free chemical and physical analyses on nanolitre- to microlitre-sized droplets suspended in air^1–12^. The suspension of droplets is typically achieved through acoustic levitation using ultrasonic waves^13–16^—the ‘acoustic tweezers’ approach to lab-on-a-drop platform design^1^. In recent years, holographic technology combining multiple low-power transducers has made it possible to control the position of objects in mid-air using ultrasound^17–21^. Macromolecular X-ray diffraction analysis^10^, Raman spectroscopy^6,7^, and DNA transfection^12^ have been conducted on acoustically levitated droplets. Contactless fluid manipulation is essential; therefore, several techniques have been proposed for droplet injection, levitation, transportation, coalescence, evaporation, and ejection^22–25^. Mixing, which is essential for diluting the sample and promoting chemical and biological reactions, is particularly challenging^26,27^: In a microlitre-sized droplet, the Reynolds number of the flow is very small (at most 2) while the Péclet number can be relatively high (10^2^–10^5^). This indicates that, despite the droplet’s small size, neither molecular diffusion nor mixing via turbulence alone can completely homogenise the components of a droplet over a realistic length of time^28^.

To prevent atmospheric contamination and changes in the concentration of the solution, the mixing time should be minimised^12,29,30^. This has been achieved by inducing sectorial oscillations at the droplet surface^22^. However, to further reduce the mixing time and expand the scope of industrial applications, the contactless-mixing mechanism in droplets needs to be explored in detail. Some experimental and theoretical investigations of this mechanism have been previously conducted^31–35^. Although a theoretical model of the interaction between molecular diffusion and mixing by advection has been proposed^31^, molecular diffusion does not seem to play a major role in reducing the mixing time. The effects of surface oscillation and internal flow have also been theoretically predicted^31^. The importance of the temporal asymmetry of modal oscillation at the surface has been clarified^33^; however, the interaction between flow and mixing has not yet been elucidated beyond the fact that the radial flow due to oscillations imposed at the surface plays a vital role in mixing^33–35^. In addition, the coalescence and mixing of droplets is generally assumed in lab-on-a-drop applications to mix multiple types of liquids. Various studies have been conducted on droplet coalescence, including internal flow, external flow, and the bridge formed between droplets^36–46^. However, to our knowledge, there are no studies investigating the flow and mixing that occurs during droplet coalescence.

Therefore, a better empirical understanding of the relationship between internal flow and droplet mixing is needed. In general, the homogenisation of components by mixing can be achieved in three ways: the stretching of fluid components by the flow field, diffusion by molecular motion, and atomisation of droplets by interfacial tension^47^. If the flow and local distribution of fluid components inside a droplet can be measured, the mechanism underlying mixing enhancement can be elucidated. However, there is a practical limitation on the simultaneous measurement of component distribution and flow field inside a droplet.

Typically, flow field and mixing measurements are carried out in separate experiments under identical conditions^48^. However, to evaluate the influence of the flow field on mixing dynamics, consistent visualization techniques are essential for measuring both velocity and concentration fields. Particle image velocimetry (PIV) is usually employed to visualize the internal flow field. There are numerous visualization techniques designed to study fluid mixing, which can be classified according to their spatial dimension of measurement. For instance, the 3D tomography methods employing gamma rays and X-rays are widely recognised, but their requirement for relatively large equipment and their insufficient spatial resolution make them unsuitable for droplets of a few millimetres in diameter, as in this study^49^. An alternative method involves the use of fluorescent dyes to gauge the degree of mixing through image processing^50^. Although this method is more cost-effective and offers superior spatial resolution, a potential issue arises when both fluorescent dyes and tracer particles are used in the same system. This could lead to interference between the luminance information of the two components. To circumvent this interference, an innovative approach could involve developing a technique to selectively separate particles within each fluid and measure the concentration field using information from the discrete particle distribution. If the particles used for PIV could also serve as markers for concentration field measurement, such a solution could be feasible. With this concept, the authors aim to develop a method that can simultaneously measure both velocity and concentration fields within a small droplet, while preserving comparable spatial and temporal resolution.

To elucidate the relationship between internal flow and mixing when oscillation is applied on the surface, herein, the surface shape, particle distributions, and flow fields in acoustically levitated droplets before and after coalescence were simultaneously analysed in detail using a novel visualisation method. The distribution of fluid elements within two droplets was visualised by selectively illuminating fluorescent particles of two different colours. This was achieved by employing red and green fluorescent particles, which were captured using a pair of synchronized high-speed cameras equipped with optical filters. Droplet coalescence and the mixing process were observed in three regimes: immediately before and after coalescence, during oscillation, and when the oscillation settled. For each timescale, the number-fraction distribution of the two types of particles was calculated to evaluate the degree of mixing. For the visualisation of flow fields, PIV was conducted for each regime to obtain velocity information about the droplets. Finally, to demonstrate the mixing performance of acoustic levitation, mixing indices were calculated on the basis of number-fraction distribution of particles. These results and the proposed method should be helpful in understanding droplet mixing dynamics and contribute to the optimisation of the acoustic-tweezers lab-on-a-drop platform.

## Results and discussions

### General observation of mixing in droplets

**Figure 1:**
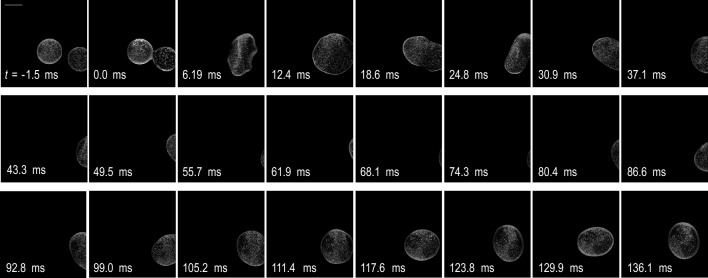
Coalescence of two ethanol droplets. Each image is a combination of images taken by two high-speed video cameras. Scale bar represents 1 mm. At time *t* = 0, two droplets start to coalesce.

Two droplets were levitated in mid-air by utilising acoustic radiation pressure, each positioned at one of two ultrasound focal points. The droplets were then purposefully merged by reducing the distance between these two focal points, resulting in a single droplet (Fig. 1). From *t* =  − 1.5 to 12.4 ms, this process could be seen in the observation window. However, owing to the displacement caused by coalescence, the droplet began to move out of the frame at *t* = 12.4–49.5 ms. It was mostly unobservable from 55.7 to 74.3 ms but returned to view at 80.4–117.6 ms.

There were bright and dark areas within the droplet owing to the fluorescence of the particles. At *t* =  − 1.5 ms, immediately before they merged, the two droplets were clearly distinguishable by their brightness difference. From 6.19 to 30.9 ms, it was still possible to distinguish the brightness distributions in the merged droplet. However, from 105.2 to 136.1 ms, the brightness distribution exhibited a complex pattern. These changes indicate that the component originating from each of the two droplets spread over the entire droplet with time. Therefore, the local concentration of each component could be quantitatively identified from the particle distribution.

The droplet achieved a distorted shape at *t* = 6.19 ms. From 12.4 to 30.9 ms, it periodically changed its elongation direction. From 99.0 to 136.0 ms, the degree of elongation gradually decreased.

### Droplet coalescence

**Figure 2:**
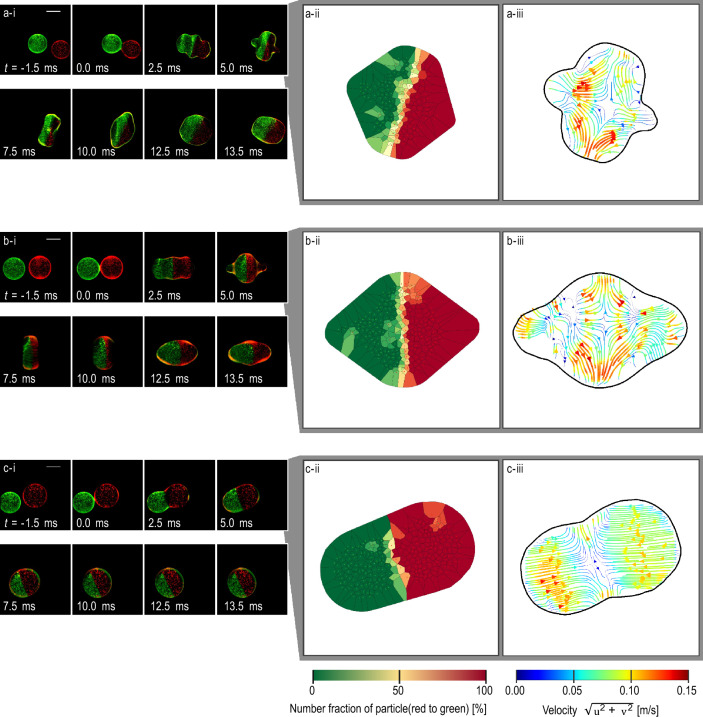
Visualisation of mixing process, number fraction of particles, and flow field in droplets immediately before and after coalescence. Coalescence of two (**a**) ethanol droplets, (**b**) 33 wt% glycerol–water solution droplets, and (**c**) pure glycerol droplets. (**i**) Photographs. (**ii**) Number fraction of particles (red and green dots) inside a droplet. (**iii**) Flow field of a droplet. For (**a-c**), (**ii**) and (**iii**) were calculated from the snapshot at *t* = 5.0 ms. Scale bar indicates 1 mm. (**a-i**), (**b-i**), and (**c-i**) correspond to Supplementary video S1, S2, and S3, respectively. At *t* = 0, two droplets start coalescing.

The behaviour of droplets immediately before and after coalescence is shown in Fig. 2. In Fig. 2-i (and in similar figures shown later in the paper), the image that captured light with a short wavelength is coloured green while the image that captured light with a long wavelength is shown in red. The surface shapes of the ethanol and glycerol-water solution droplets were distorted after coalescence. On the other hand, the surface shape of the pure glycerol droplet remained circular. From Fig. 2a-i, at *t* = −1.5 ms, it can be seen that the two component droplets were clearly distinguishable by the red and green particles. During coalescence, at *t* = 2.5 ms, the green and red particles were distributed on the left and right sides, respectively, of the droplet. Thus, the components of the droplet could be visualized using the two monochrome cameras and optical filters.

The distribution of the particle-number fraction at *t* = 5.0 ms is shown in Fig. 2-ii for each case in a-c. As shown in Fig. 2-i, until *t* = 13.5 ms, the green and red particles were distributed on the left and right sides, respectively, of each droplet. This was reflected in the number-density distributions (except in the middle of each droplet, i.e., the contact area of the two original droplets, wherein cells with number fractions close to 50% were predominant). Therefore, both the images of the particles detected and number-fraction distributions reflected the expected distribution of particles.

Figure 2-iii shows the velocity field inside each droplet. In all three cases, coalescence produced opposing horizontal flow. Moreover, the flow sometimes changed direction from left–right to up–down. Considering the similarity of this to the case of a droplet with surface oscillation induced^22,35^, the flow was probably caused by the deformation of the surface.

The maximum velocity in Fig. 2a-iii and b-iii was 0.15 m/s while that in Fig. 2c-iii was 0.10 m/s. Therefore, the maximum velocity was different for droplets with different compositions.

### Surface oscillation of the droplet

**Figure 3:**
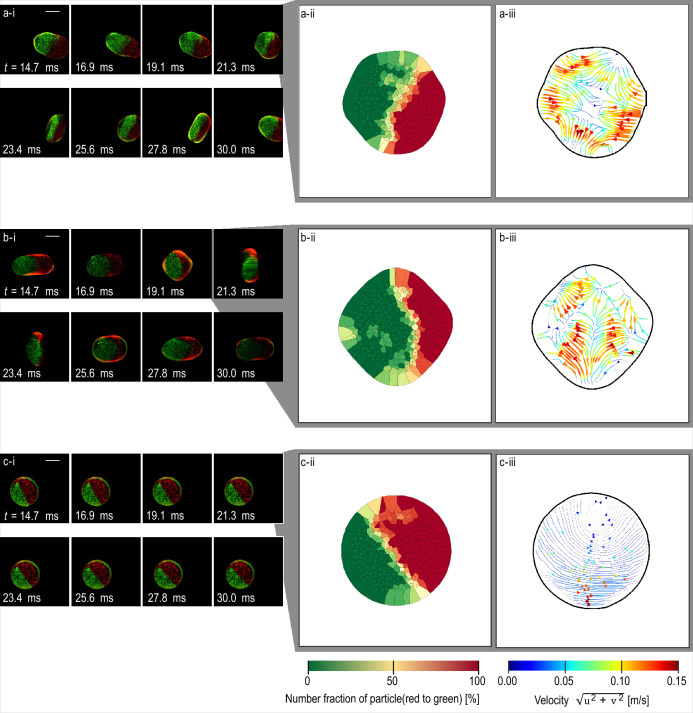
Visualisation of mixing process, number fraction of particles, and flow field in droplets during oscillation. Droplets formed from (**a**) two ethanol droplets, (**b**) two 33 wt% glycerol–water solution droplets, and (**c**) two pure glycerol droplets. (**i**) Photographs. (**ii**) Number fraction of particles (red and green dots) inside a droplet. (**iii**) Flow field of a droplet. For (**a**) and (**c**), (**ii**) and (**iii**) were calculated from the snapshot at *t* = 19.1 and 21.3 ms. For (**b**), (**ii**) and (**iii**) were calculated from the snapshot at *t* = 19.1 ms. Scale bar indicates 1 mm. At *t* = 0, two droplets start coalescing. (**a-i**), (**b-i**), and (**c-i**) correspond to Supplementary video S4, S5, and S6, respectively.

The behaviour of the droplets from *t* = 14.7 ms to 30.0 ms is shown in Fig. 3. The direction of elongation changed periodically (Fig. 3a-i and b-i). By contrast, there was no deformation of the pure glycerol droplet (Fig. 3c-i). The time dependence of the amplitude, *A*, of surface oscillation of the droplets is^51^

1 $$\begin{aligned} & A\propto {e}^{-\frac{t}{{\tau }_{n}}},\\& {\mathrm{where}}\; \frac{1}{{\tau }_{n}}=\frac{\nu }{{r}^{2}}\left(n-1\right)\left(2n+1\right),\end{aligned}$$

where *t* represents the time, ν, the kinematic viscosity, *r*, the volume-equivalent radius, and *n*, the mode of oscillation. Thus, increasing the viscosity of the droplets also increases the damping effect of surface oscillation. It can be seen from Fig. 3-i that for the 2nd mode oscillation, the width of each droplet was 2–3 mm. Owing to the acoustic radiation pressure, they were oblate horizontally; therefore, the height had a weaker effect on their volume than width. Thus, the droplets in this study were not expected to have significant differences in volume or volume-equivalent diameter *R*. In contrast, under the experimental conditions, the kinematic viscosities were 1.387 × 10^−6^, 2.715 × 10^−6^, and 720.0 × 10^−6^ m/s for ethanol, 33 wt% glycerol–water solution, and pure glycerol, respectively. Therefore, the damping effect of surface oscillation was twice as strong for the 33 wt% glycerol–water solution and 720 times stronger for pure glycerol than that for ethanol. These results confirmed that the difference in surface oscillation during coalescence was due to the difference in the droplets’ kinematic viscosities.

The distribution of the particle-number fraction at *t* = 21.3 ms (Fig. 3-ii) was different from that at *t* = 5.0 ms (Fig. 2-ii). For all three cases in Fig. 3-i, although the green and red particles were concentrated on the left and right sides, respectively, of the droplet, the area with number fraction close to 50% was much larger than that observed in Fig. 2. This indicates that the mixing progressed with time.

Figure 3-iii shows the velocity field inside the droplets. A radial flow still existed in ethanol and the 33 wt% glycerol–water solution (Fig. 3a-iii and b-iii, respectively), and the maximum velocity of the flow was 0.15 m/s for both droplets. However, in the pure glycerol droplet shown in Fig. 3c-iii, there was only circumferential flow caused by the rotation of the droplet. Therefore, the velocity of the flow decreased near the centre. In a previous study^25,35^, a relationship was found between the surface oscillation and internal flow of a droplet. Thus, in this study, the internal flow of the droplet was attributable to the oscillation at the surface.

### Stable levitation after oscillation

**Figure 4:**
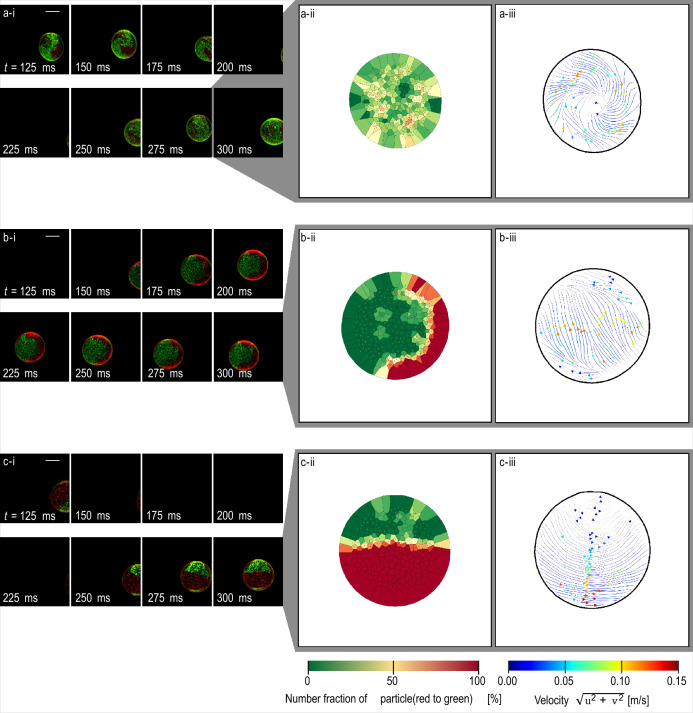
Visualisation of mixing process, number fraction of particles, and flow field in droplets after oscillation. Droplet formed from (**a**) two ethanol droplets, (**b**) two 33 wt% glycerol–water solution droplets, and (**c**) two pure glycerol droplets. (**i**) Photographs. (**ii**) Number fraction of particles (red and green dots) inside a droplet. (**iii**) Flow field of a droplet. For (**a**), (**ii**) and (**iii**) were calculated from the snapshot at* t* = 275 and 300 ms. For (**b**) and (**c**), (**ii**) and (**iii**) were calculated from the snapshot at *t* = 300 ms. Scale bar indicates 1 mm. At *t* = 0, two droplets start coalescing. (**a-i**), (**b-i**), and (**c-i**) correspond to Supplementary video S7, S8, and S9, respectively.

The behaviour of the droplet after surface oscillation reached steady state is shown in Fig. 4. In ethanol (Fig. 4a-i), the distribution of the green and red particles was more complex at *t* = 125 ms than at *t* = 21.3 ms (Fig. 3a-ii), and the complexity increased as time passed. Finally, at *t* = 300 ms, the two types of particles were randomly distributed. In contrast, in the 33 wt% glycerol–water solution and pure glycerol (Fig. 4b-ii and c-ii, respectively), the two types of particles were still separated even at *t* = 300 ms. Specifically, in the pure glycerol droplet (Fig. 4c-ii), they remained segregated into opposite hemispheres.

The surface deformation still existed in the ethanol droplet at *t* = 175 ms shown in Fig. 4a-i. In contrast, the droplets of the 33 wt% glycerol–water solution and pure glycerol (Fig. 4b-i and c-i, respectively) were nearly spherical.

Different rates of mixing were also reflected in the number-fraction distribution of the particles shown in Fig. 4-ii. For ethanol (Fig. 4a-ii), cells with number fractions close to 50% were observed throughout the entire droplet. However, for the 33 wt% glycerol–water solution (Fig. 4b-ii), cells with number fractions close to 50% had a bow-shaped distribution in the lower right corner of the droplet; the change in the distribution was due to the internal flow. Finally, for pure glycerol (Fig. 4c-ii), the distribution was the same as that observed at *t* = 21.3 ms shown in Fig. 3c-ii.

Finally, Fig. 4-iii shows the velocity field inside each droplet. For all three cases, there was only circumferential flow.

### Quantification of mixing performance

**Figure 5:**
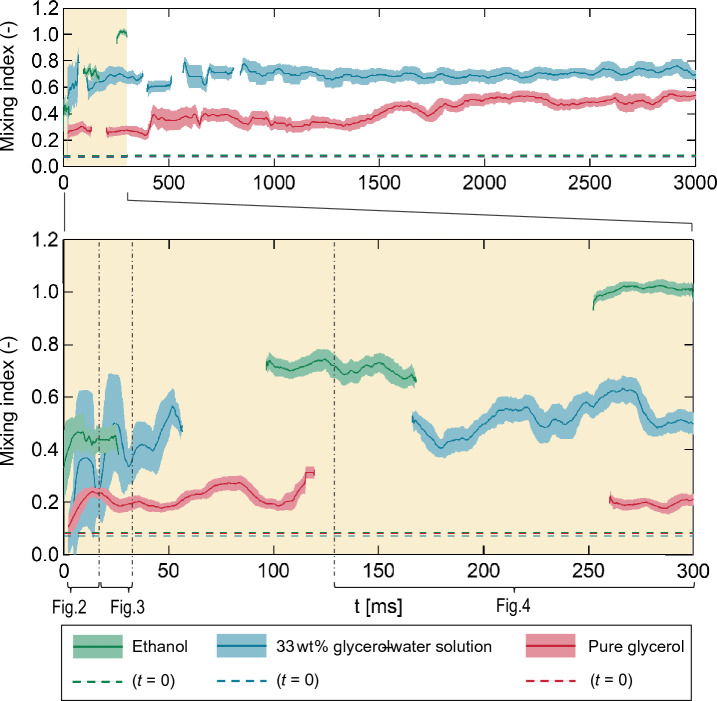
Time evolution of mixing index *Mc* after droplet coalescence. Lower figure shows the first 300 ms of the upper figure. Each solid line shows the moving average of 50 frames; the error band shows the standard deviation. Interruption in the lines correspond to when the droplet was outside the imaging area. Dashed lines correspond to the initial *Mc*’s when the droplets coalesced at *t* = 0.

To quantify the degree of mixing, the Lacey mixing index, *Mc*^52,53^, was calculated for each droplet from the number-fraction distribution (Fig. 5); *Mc* = 0.0 corresponds to a completely separated state while *Mc* = 1.0 corresponds to a completely mixed one. For all three droplets, *Mc* < 0.1 immediately after coalescence. The *Mc* of ethanol was very close to 1 at 300 ms, which indicates that the two components originating from separate ethanol droplets had become randomly distributed. In contrast, the *Mc*’s of the glycerol–water solution and pure glycerol droplets were 0.5 and 0.2, respectively, at 300 ms. This trend was qualitatively similar to that observed for the degree of mixing after coalescence, at 300 ms (Fig. 4). In addition, all three *Mc*’s increased with time. This indicates that the *Mc*’s reflected the actual mixing states of the component droplets, i.e., the degree of mixing was successfully quantified.

For ethanol, *Mc* increased from 0.1 to 0.4 until *t* = 30 ms, then gradually increased from 0.4 to 1.0 until *t* = 300 ms. For *t* < 30 ms, the surface exhibited significant oscillation (Figs. 2a-i and 3a-i). Although surface oscillation subsided with time, it still existed at *t* = 175 ms, as shown in Fig. 4a-i.

For the 33 wt% glycerol–water solution droplet, *Mc* increased from 0.1 to 0.5 until *t* = 50 ms, after which it stayed relatively constant. As in the case of ethanol droplets, the surface also exhibited significant oscillation for *t* < 30 ms (Figs. 2a-i and 3a-i). However, surface oscillation of the interface was negligible at *t* = 175 ms (Fig. 4b-i).

Finally, for the pure glycerol droplet, *Mc* increased from 0.1 to 0.2 until *t* = 10 ms, maintaining that value thereafter. From *t* = 0.0 to 7.5 ms, the contact area of the original droplet component increased (Fig. 2c-i). Thereafter, no oscillation was observed at the surface (Figs. 2c-i, 3c-i, 4c-i).

The contrasting observations made between the *Mc* and droplet behaviour suggests three possible factors that led to the increase the *Mc*. The first factor was the increase in the contact area of the original droplet components due to coalescence of the droplets. In this study, no oscillation was observed at the surface of pure glycerol droplet. However, a comparison of Fig. 5 with Fig. 2c-i shows that for the pure glycerol droplet, *Mc* increased from 0.1 to 0.2 as the two droplets coalesced into a single droplet from *t* = 0.0 to 7.5 ms. The second factor was the intensity of surface oscillation: for *t* < 30.0 ms (Figs. 2 and 3), oscillation at the surface were present in the ethanol and 33 wt% glycerol–water solution droplets, with *Mc* = 0.4 at around *t* = 30.0 ms. In contrast, surface oscillation was absent in the pure glycerol droplet with *Mc* = 0.2. The final factor was the duration of surface oscillation. Specifically, at *t* = 300.0 ms, *Mc* = 1.0 for the ethanol droplet, for which oscillations at the surface were still observed at *t* = 175 ms. By contrast, *Mc* = 0.5 for the 33 wt% glycerol–water solution droplet, for which surface oscillation was no longer observed at *t* = 125 ms. Therefore, the aforementioned three factors are important for droplet mixing.

### Summary

In this study, the fluid distribution and internal flow were measured simultaneously to understand the mixing dynamics of acoustically levitated airborne droplets. To accomplish this, we have developed a technique that selectively separates fluorescent particles within each fluid, and measures the concentration field based on the information derived from the discrete particle distribution. The proposed method presents a valuable tool for probing the relationship between the flow field and mixing, particularly for contactless quantitative study of physicochemical phenomena. Furthermore, it can significantly enhance our understanding of unsteady-state fluid dynamics in scenarios with time-induced volume and concentration variations.

Although we identified the relationship between surface oscillation and degree of mixing of acoustically levitated droplets. The present results provide insights into the dynamics of acoustically levitated airborne droplets and will be useful in the study of microfluidic interfacial dynamics, flow fields, heat/mass transfer, and chemical reactions. Such investigations will allow the contactless selection and manipulation and optimal sample handling for future lab-on-a-drop applications.

## Methods

### Experimental setup

**Figure 6:**
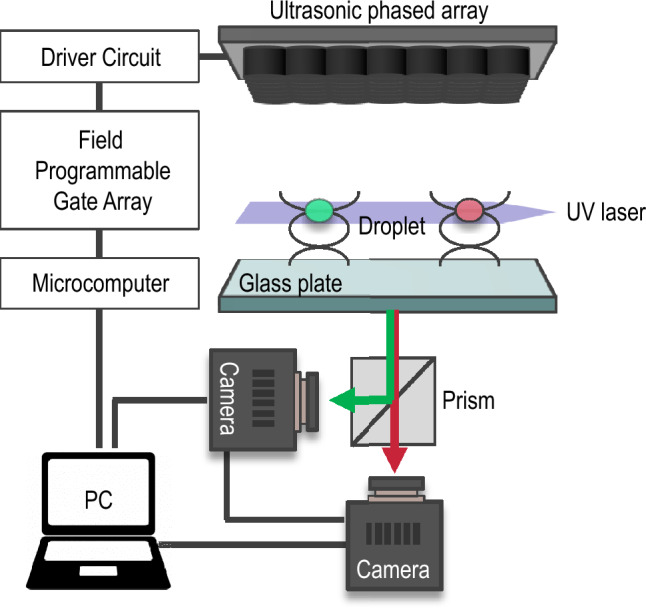
Schematic of experimental setup.

Acoustic levitation was implemented in this study using an ultrasonic phased array^17^. The focal points of sound can be generated at arbitrary positions by transmitting sound waves with a controlled phase; localised acoustic standing waves can then be generated near the focal points using a glass plate. We employed a 7 × 7 square transducer array consisting of 49 small ultrasonic transducers (Nippon Ceramic Co., Ltd, T4010B4). The diameter and frequency of the transducers were 10 mm and 40 kHz, respectively. To generate ultrasonic focal points, the phase of the sound transmitted from each transducer was controlled using a field-programmable gate array (FPGA) (Altera Co., Cyclone-IV DE0-Nano). Figure 6 shows the experimental setup. The focal length and distance from the transducer to the reflector were both 40 mm. Two droplets could be levitated by switching the focal points at a frequency of 500 Hz^17^. The levitated droplets can be transferred and coalesced by actively controlling the distance between the focal points^22^. The ambient temperature and relative humidity were 25.0 ± 0.5 ℃ and 40 ± 10%, respectively. We used ethanol, 33 wt% glycerol–water solution, and pure glycerol to compare the effects of kinematic viscosity on the flow characteristics of a droplet with surface oscillation.

**Figure 7:**
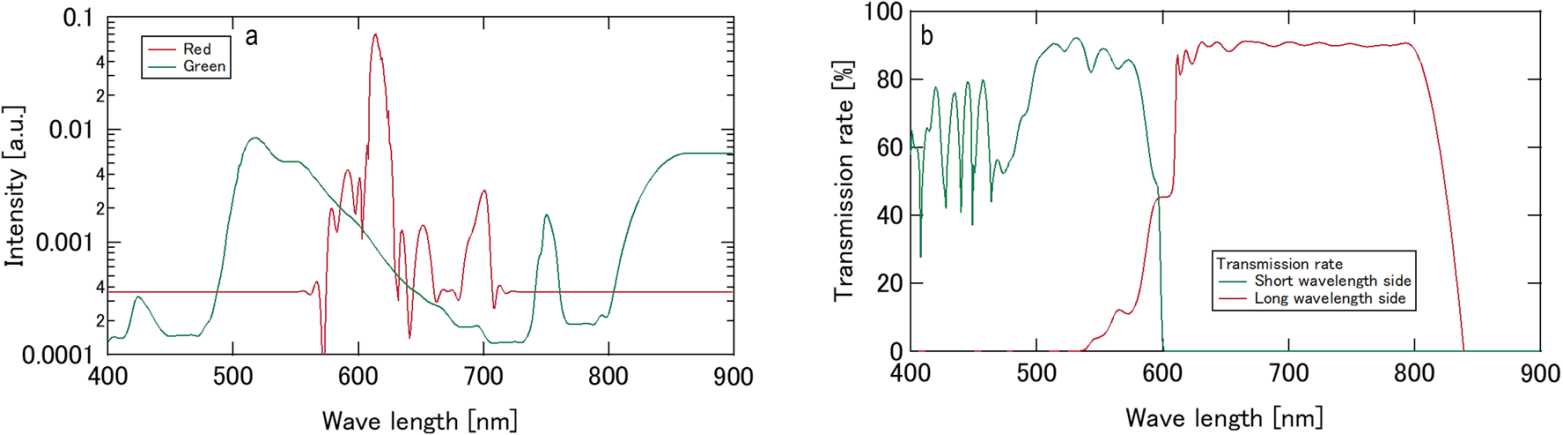
Characteristics of fluorescence and transmittance. (**a**) Fluorescent of tracer particle. (**b**) Transmittance of optical system.

To visualise the flow and concentration fields inside the droplets, we employed red and green fluorescent acrylic particles from Central Techno Co. Ltd., known as Lumisis markers. These particles have an average diameter of 10 μm and a specific weight of 1.19. In comparison, the specific weights for ethanol, a 33 wt% glycerol–water solution, and pure glycerol are 1.51, 1.11, and 0.94 respectively. The density of the particles was suitably compatible with the fluid densities used in our study. The concentrations of both red and green particles were meticulously controlled to ensure they were identical. A UV sheet laser from Central Techno Co. Ltd. with a wavelength of 375 nm and a maximum power of 350 mW, was used to irradiate the cross-sections of droplets containing these fluorescent particles. The fluorescence characteristics are shown in Fig. 7(a). The behaviour of the two levitated droplets was monitored from below using a pair of synchronised monochromatic high-speed video cameras from Photron Co., Ltd. (FASTCAM-Mini AX200). These cameras were positioned on the short and long wavelength sides. Additionally, each camera was equipped with a short-pass filter (from THORLABS Co. Ltd., FESH0600) and a long-pass filter (from Kenko Co. Ltd., YA3). Figure 7(b) shows the transmittance of the optical system. To differentiate between the red and green fluorescent light emitted from the particles, a dichroic prism and optical long and short pass filters were employed. The frame rate was set at 6400 fps, with an exposure time of 1/6400 s.

In an effort to ascertain whether the mixing time of a solution can be observed through the use of particles in our study—particles that are several orders of magnitude larger than the size of molecules—we explored the mixing dynamics by considering physical timescales.

**Figure 8:**
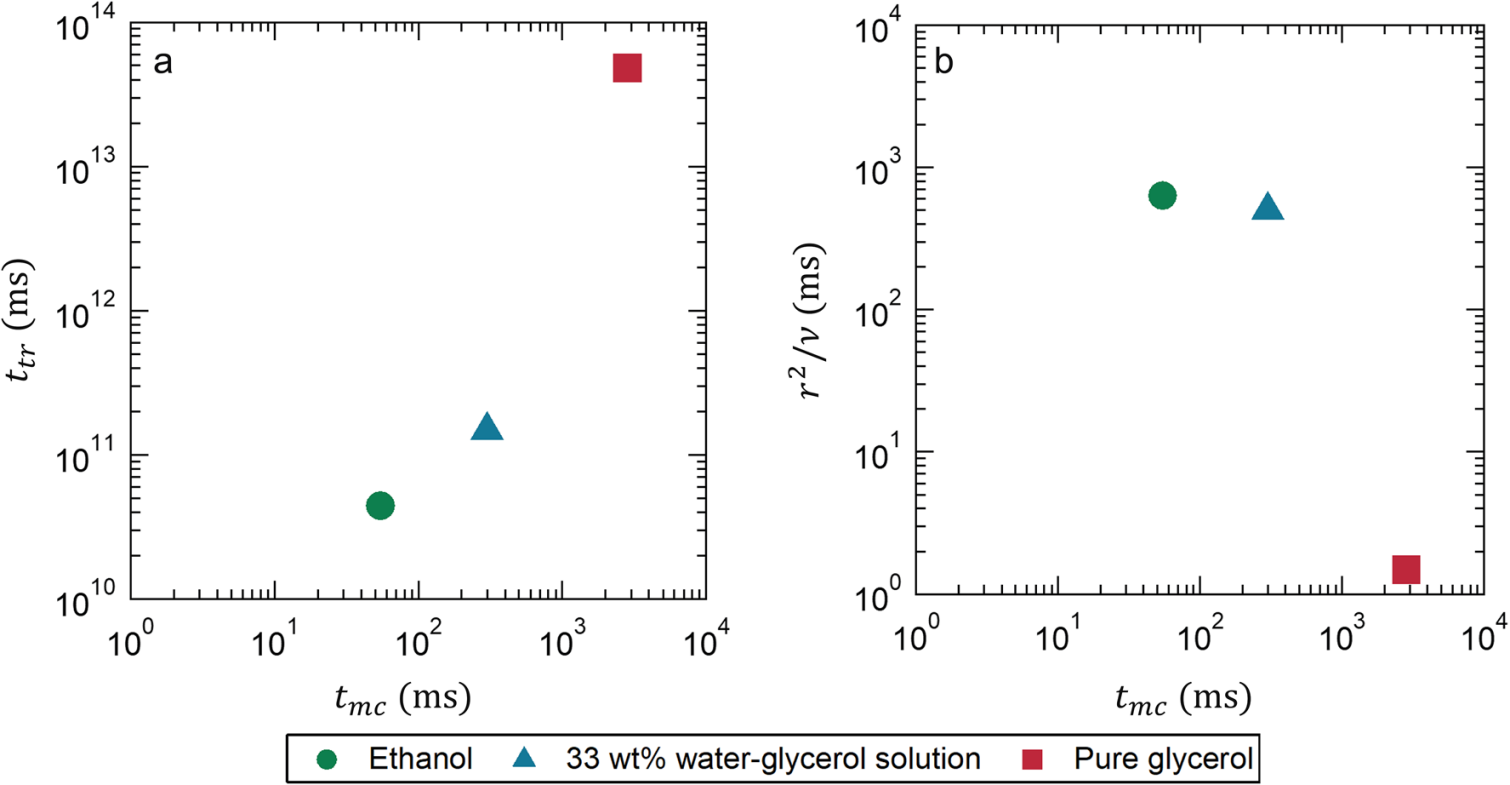
Comparison of physical timescales considered for droplet mixing. The mixing time *t*_*mc*_ for *Mc* = 0.5 (as shown in Fig. 5b) is compared with (**a**) the particle diffusion time *t*_*tr*_ and (**b**) the viscous dissipation time, both as functions of the droplet radius *r* and the kinematic viscosity *ν*.

Figure 8 presents a comparison of these physical timescales considered for the process of droplet mixing. Initially, we introduced the diffusion timescale of the tracer particle, which is computed as follows:

2 $$\begin{aligned}{t}_{tr}=\frac{1}{{D}_{tr}}{\left(\frac{d}{2}\right)}^{2},\\ {\mathrm{where}}\; {D}_{tr}=\frac{{k}_{B}T}{6\pi \eta {r}_{p}}\end{aligned}$$

where *d* is the equivalent diameter of droplets, *k*_*B*_ the Boltzmann constant, *T*, the absolute temperature, *η*, the viscosity of liquid, and *r*_*p*_, the radius of the tracer particle. Figure 8a compares *t*_*mc*_ and *t*_*tr*_; for all cases, *t*_*tr*_ $\gg$ *t*_*mc*_. This indicates that the mixing of tracer particles in the droplet was amplified by the internal flow resulting from droplet coalescence.

The internal flow was driven by the momentum transfer within the droplet, leading us to choose the viscous dissipation timescale as the second timescale for consideration. Figure 8b illustrates the relationship between *t*_*mc*_ and *r*^2^/*ν.* It is evident that *t*_*mc*_ decreases with a reduction in kinematic viscosity (or an increase of *r*^2^/*ν*). This suggests that momentum transfer was enhanced for droplets with lower viscosity, leading to a reduction in *t*_*mc*_ due to the impact of internal flow. Based on these findings, it can be concluded that advection is superior to molecular diffusion in terms of the mixing timescale in all cases we examined. As advection plays a pivotal role in solution mixing in our study, our methodology—predicated on particle colour—facilitates the measurement of concentration within localized areas of a droplet.

### Image processing

For the visualisation of the mixing processes, images were developed by the following steps: (1) preparation of a set of pictures with dotted patterns; (2) calculation of the distortion and skew of images; (3) creation of correction functions for the distortion of each camera; (4) correction of image distortion for every picture obtained in the experiments; and (5) composition of the pictures into a single image.

For flow visualisation in the droplets, PIV was conducted using Koncerto II (Seika Digital Image Co., Japan).

### Calculation of mixing index

**Figure 9:**
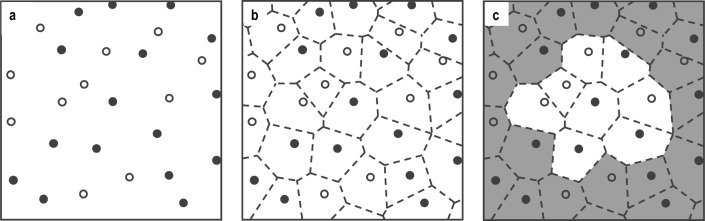
Procedure for calculating mixing index. (**a**) Detection of particles in image. (**b**) Corresponding Voronoi diagram. (**c**) Particles adjacent to central black particle. White or black circles represent red or green particles.

To quantify the component distribution, an objective mixing index must be defined based on the number-density distribution of the particles. A schematic of the calculation procedure is shown in Fig. 9. First, the particles were detected from images taken by the high-speed video cameras (Fig. 9a). Next, the number fractions of neighbouring particles were used to calculate the number-fraction distribution, and a Voronoi diagram was developed accordingly, as shown in Fig. 9b. The particles were assumed to be adjacent when their respective Voronoi cells were in contact with each other (Fig. 9c). The number-fraction distribution in each cell was calculated as follows:

3 $${\theta }_{p,j}=\frac{{N}_{p,j}}{{\sum }_{p}{N}_{p,j}},$$

where *θ* is the number fraction, *N*, the number of neighbouring particles, *p*, the species of the particle, and *j*, the index of each particle.

To quantify the mixing state, we calculated *Mc*^52,53^:

4 $${M}_{C}={\sum }_{p}\frac{{N}_{p}}{{\sum }_{p}{N}_{p}}{M}_{c,p}, {M}_{c,p}=\frac{{s}_{p}^{2}-{s}_{p,0}^{2}}{{s}_{p,R}^{2}-{s}_{p,0}^{2}},$$

where

5 $$\begin{aligned}{s}_{p}^{2}=\langle {\left({\theta }_{p,j}-\langle {\theta }_{p,j}\rangle \right)}^{2}\rangle,\\ {s}_{p,0}^{2}=\langle {\theta }_{p,j}\rangle \left(1-\langle {\theta }_{p,j}\rangle \right),\\ {s}_{p,R}^{2}=\frac{\langle {\theta }_{p,j}\rangle \left(1-\langle {\theta }_{p,j}\rangle \right)}{\langle {n}_{j}\rangle }.\end{aligned}$$

Here, the number-fraction distribution *θ*_*p,j*_ is the local concentration of species *p* around particle *j*; *n*_*j*_ is the number of neighbouring particles around particle *j*; and* s*_*p*_^2^*, s*^2^_*p,0*_, and* s*^2^_*p,R*_ are the variances of the local particle-number fractions: *s*_*p*_^2^ is the variance at a given time, *s*^2^_*p,0*_, the variance at the initial time when the fluids were completely separated, and *s*^2^_*p,R*_, the variance at a time when the fluids were sufficiently mixed.

### Supplementary Information

Supplementary Video S1.

Supplementary Video S2.

Supplementary Video S3.

Supplementary Video S4.

Supplementary Video S5.

Supplementary Video S6.

Supplementary Video S7.

Supplementary Video S8.

Supplementary Video S9.

## Data Availability

The datasets generated and/or analysed during the current study are available from the corresponding author on reasonable request.
